# A novel alternatively spliced isoform of the mu-opioid receptor: functional antagonism

**DOI:** 10.1186/1744-8069-6-33

**Published:** 2010-06-02

**Authors:** Pavel Gris, Josee Gauthier, Philip Cheng, Dustin G Gibson, Denis Gris, Oskar Laur, John Pierson, Sean Wentworth, Andrea G Nackley, William Maixner, Luda Diatchenko

**Affiliations:** 1 Center for Neurosensory Disorders, University of North Carolina at Chapel Hill, Chapel Hill, NC, 27599, USA; 2 Lineberger Comprehensive Cancer Center, University of North Carolina at Chapel Hill, Chapel Hill, NC, 27599, USA; 3 Yerkes Research Center - Division of Microbiology, Emory University, Atlanta, GA, 30329, USA

## Abstract

**Background:**

Opioids are the most widely used analgesics for the treatment of clinical pain. They produce their therapeutic effects by binding to μ-opioid receptors (MORs), which are 7 transmembrane domain (7TM) G-protein-coupled receptors (GPCRs), and inhibiting cellular activity. However, the analgesic efficacy of opioids is compromised by side-effects such as analgesic tolerance, dependence and opioid-induced hyperalgesia (OIH). In contrast to opioid analgesia these side effects are associated with cellular excitation. Several hypotheses have been advanced to explain these phenomena, yet the molecular mechanisms underlying tolerance and OIH remain poorly understood.

**Results:**

We recently discovered a new human alternatively spliced isoform of MOR (MOR1K) that is missing the N-terminal extracellular and first transmembrane domains, resulting in a 6TM GPCR variant. To characterize the pattern of cellular transduction pathways activated by this human MOR1K isoform, we conducted a series of pharmacological and molecular experiments. Results show that stimulation of MOR1K with morphine leads to excitatory cellular effects. In contrast to stimulation of MOR1, stimulation of MOR1K leads to increased Ca^2+ ^levels as well as increased nitric oxide (NO) release. Immunoprecipitation experiments further reveal that unlike MOR1, which couples to the inhibitory Gα_i/o _complex, MOR1K couples to the stimulatory Gα_s _complex.

**Conclusion:**

The major MOR1 and the alternative MOR1K isoforms mediate opposite cellular effects in response to morphine, with MOR1K driving excitatory processes. These findings warrant further investigations that examine animal and human MORK1 expression and function following chronic exposure to opioids, which may identify MOR1K as a novel target for the development of new clinically effective classes of opioids that have high analgesic efficacy with diminished ability to produce tolerance, OIH, and other unwanted side-effects.

## Background

The μ-opioid receptor (MOR) is the primary target for both endogenous and exogenous opioid analgesics, mediating basal nociception as well as agonist responses [1-4]. While opioids are the most frequently used and effective analgesics for the treatment of moderate to severe clinical pain, their prolonged use leads to a number of adverse side-effects, including tolerance, dependence, and post-dosing induced hyperalgesia, which is commonly referred to as "opioid-induced hyperalgesia" (OIH) [5-7]. Several hypotheses have been advanced to explain the mechanisms underlying tolerance and OIH, including opioid receptor downregulation, receptor desensitization, and/or a decreased efficiency in G protein coupling. The currently held hypotheses fail to fully explain the mechanisms that contribute to tolerance and OIH. For example, receptor downregulation does not parallel the development of tolerance to opioids [8]. Additionally, the desensitization of opioid receptor signaling following repeated or prolonged opioid treatment [9] is unlikely to account for opioid-induced tolerance as it has been reported to suppress the development of tolerance [10]. Thus, the molecular mechanisms underlying opioid tolerance and OIH require further investigation. One important, yet underemphasized, cellular consequence of chronic opioid treatment is the unmasking of excitatory signaling and the suppression of the canonical inhibitory signaling pathways [11-13].

The canonical signaling pathway for MOR agonists is facilitated through a pertussis toxin (PTX)-sensitive inhibitory G protein (Gα_i/o_), where analgesia reflects the inhibition of synaptic transmission via inhibition of presynaptic and postsynaptic voltage-gated Ca^2+ ^channels (VGCC) and/or a decrease in neuronal excitability *via *activation of inwardly rectifying K^+ ^channels. While opioid-induced regulation of K^+ ^current in sensory neurons [14] and inhibition of adenyl cyclase (AC) have been implicated in suppressing the activity of pronocicepitve sensory primary neurons [15,16], the VGCC appears to be the primary target underlying rapid opioid mediated effects in these neurons [17,18]. This rapid inhibition of VGCC reflects both a voltage-dependent and -independent inhibition of high threshold channels[19-22]. MOR-mediated inhibition of VGCC on central presynaptic terminals of primary afferent nociceptors is thought to be one of the primary mechanisms mediating analgesia at the spinal level. However, opioid-induced hyperalgesic responses have also been shown in animals and man following both acute and chronic dosing [23-26]. These hyperalgesic effects are associated with concentration- and time-dependent cellular excitation [15,16,27] as well as with biphasic effects on cAMP formation and Substance P release [13,16,27-30]. Available evidence suggests these excitatory effects reflect the activation of a stimulatory G protein (Gα_s_) [11,31].

Using new bioinformatic approaches, we have recently established the existence of previously undetected exons within the human μ-opioid receptor gene *OPRM1 *[32]. These exons were discovered in a human genetic association study that identified several single nucleotide polymorphisms (SNPs) associated with the individual variability in pain sensitivity and responses to the MOR agonist morphine. We found that exons carrying these functional SNPs are spliced into a *OPRM1 *variant named *MOR1K *that encodes for a 6TM rather than a canonical 7TM G-protein coupled receptor. The extracellular N-terminus and first cytoplasmic domain are missing from this isoform. Instead, MOR1K possesses a cytoplasmic N-terminus followed by 6 transmembrane domains and C-terminus homologous to MOR1. Thus, MOR1K should retain the ligand binding pocket that is distributed across the conserved TMH2, TMH3, and TMH7 domains [33] and be capable of binding MOR agonists. Genetic analyses revealed that allelic variants coding for higher MOR1K expression are associated with greater sensitivity to noxious stimuli and blunted responses to morphine[32]. This relationship is opposite to that expected for MOR and suggests a pronociceptive function for MOR1K. We thus hypothesized that MOR1K contributes to hyperalgesic effects of MOR agonists through the activation of cellular excitatory pathways. To test this hypothesis, we first characterized tissue-specific expression levels of MOR1K, its cellular localization, and agonist binding capacity to confirm potential functionality of this new receptor isoform. We then employed pharmacologic and molecular biologic methods to measure and compare intracellular cAMP and Ca^2+ ^levels as well as nitric oxide (NO) release in response to stimulation of canonical MOR1 and alternative MOR1K isofoms. Immunoprecipitation experiments were carried out to test if the MOR1K isoform couples to the stimulatory Gα_s _complex.

## Results And Discussion

### MOR1K expression and binding pattern

**Figure 1:**
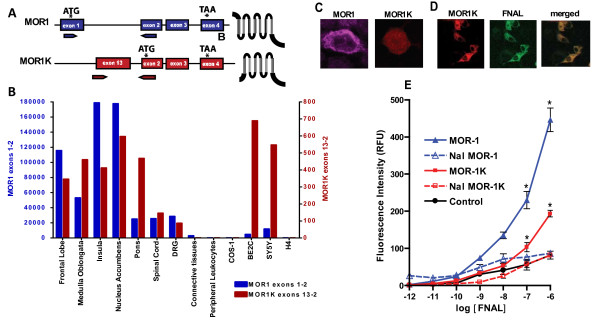
**MOR1K expression and binding pattern**. (A) The schematic diagram illustrates the exonic composition and relative positions of PCR primers designed to amplify the major *MOR1 *isoform and the newly identified alternative *MOR1K *isoform. The relative positions of translation initiation start and stop codons are designated by ATG and TGA, respectively. The predicted protein structure of *MOR1 *and *MOR1K *isoforms is schematically presented. Translation of the *MOR1K *variant results in a 6TM receptor, truncated at the N-terminus. (B) Real-time PCR was performed on total RNA samples from the human brain regions known to express *MOR1*. Primers specific for exons 1 and 2 were used to measure MOR1 and primers specific for exons 13 and exon 2 were used to measure *MOR1K*[32]. GAP3DH was used as a control for cDNA loading and PCR efficiency. (C) Confocal images of C-terminally MYC-tagged MOR1 or FLAG-tagged MOR1K overexpressed in HEK293 cells and stained with either Anti-MYC-Tag Antibody (Alexa Fluor 647 Conjugate) or Anti-DYKDDDDK Tag Antibody (Alexa Fluor 555 conjugate). Cells transfected with MOR1 showed membrane expression of receptor, while cell transfected with MOR1K express receptor only intracellular. (D) Confocal images of C-terminally FLAG-tagged MOR1K overexpressed in Be2C cells and stained with either Anti-FLAG M2 Antibody Alexa Fluor Conjugate or fluorescent-labeled naloxone (FNAL). Cells transfected with MOR1K showed intracellular retention of FNAL that co-localized with antibody-labeled receptor. (E) The binding of naloxone to MOR1K was assessed using flow cytometry to measure FNAL retention. Be2C cells transfected with either MOR1 or MOR1K isoforms showed increased retention of FNAL at concentrations of 0.1 and 1 μM. FNAL retention was abolished in the presence of 10 μM unlabelled naloxone (Nal). In panel E, data are presented as mean + SEM. *P < 0.05 different from controls).

First, we characterized the relative expression of *MOR1K *in human brain and spinal cord as well as peripheral leukocytes using real-time PCR (RT-PCR). Results from RT-PCR revealed that MOR1K is expressed in the frontal lobe, medulla oblongata, insula, nucleus accumbens, pons, spinal cord, and dorsal root ganglion (DRG) (Fig.1A-B). These brain regions are known to also express MOR1 and contribute to the pharmacological effects of MOR agonists. MOR1K expression was not observed in the connective tissue surrounding DRG or in peripheral leukocytes. Additionally, MOR1K was expressed at high levels in the human neuroblastoma cell lines Be2C and SH-SY5Y, while no expression was observed in monkey kidney COS-1 and human astrocytoma H4 cell lines. These data suggest that human *MOR1K *expression is restricted to neuronal cells. As the highest relative expression levels are in transformed neuronal cell lines, *MOR1K *expression is likely to be suppressed in native cellular conditions.

To characterize the cellular location and function of the new human MOR1K isoform, expression vectors with cloned coding regions of MOR1 (7TM) or MOR1K (6TM) receptor isoforms, with or without MYC (MOR1) or FLAG (MOR1K) tags at their N-termini, were transiently transfected into a cell line that expresses endogenous MORs (i.e., Be2C human neuroblastoma cell line) and a cell line that does not express endogenous MORs (i.e., african green monkey kidney COS1 and human embryonic kidney (HEK293) cells). Overexpression of MOR1K in mammalian cells revealed that this 6TM receptor is not expressed at the cell membrane, but instead is retained in the intracellular compartment (Fig.1C). As intracellular localization of several receptor systems does not prevent receptor-mediated signalling [34,35], we examined whether the intracellulary localized MOR1K binds MOR ligands using flow cytometry [36] to measure binding of fluorescently labeled naloxone (Fig.1D-E). Be2C cells transfected with either MOR1 or MOR1K retained a significantly higher proportion of fluorescently-labeled naloxone in comparison with cells transfected with an empty vector control. This retention was abolished in the presence of excess of unlabelled naloxone demonstrating the specificity of binding. These data suggest that MOR ligands, such as naloxone, can cross the plasma membrane and MOR1K is a functional intracellular receptor that binds MOR ligands. Although this set of studies has employed naloxone, a movement of morphine across the cellular membrane by means of active transport or passive diffusion has also been shown [37,38]. Furthermore, the rate of passive diffusion of weak base ligands like morphine across the cell membrane can be examined by the Henderson-Hasselbalch equation [39,40], estimating that approximately 25% of the total amount of morphine present in the medium can diffuse across membranes to enter or exit cells.

### Effect of MOR1K activation on cAMP, Ca^2+ ^and NO levels

**Figure 2:**
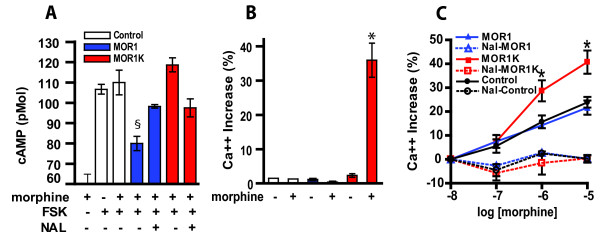
**MOR1K activation stimulates cAMP and Ca^2+^**. COS1 (A,B) or BE2C (C) cells were transiently transfected with MOR1K, MOR1, or empty vector control expressing constructs. (A) Forskolin (FSK, 10 μM) was used to increase cAMP levels prior to morphine treatment. Following morphine treatment, cells expressing MOR1 exhibited reduced cAMP levels, while those expressing MOR1K did not. In fact, cells expressing MOR1K exhibited a trend towards an increase in cAMP levels. (B) Following morphine treatment, COS1 cells expressing MOR1K exhibited substantial increases in Ca^2+ ^levels, while those expressing MOR1 did not. (C) Stimulation of MOR1K with morphine produced a robust dose-dependent increase in Ca^2+ ^levels in Be2C cells. This increase was significantly different from the moderate increase in morphine-evoked Ca^2+ ^levels observed in Be2C cells transfected with MOR1 or empty vector, which were likely due to the high endogenous expression of MOR1K in Be2C cells. For all panels, both MOR1 and MOR1K, morphine-dependent effects were antagonized in the presence of naloxone (0.1 μM). Data are presented as mean ± S.E.M from at least 6 experiments. $*P *< 0.05 different from control and *P < 0.05 different from control and MOR1.

Next, we characterized the cellular effects of MOR1K stimulation using cAMP, Ca^2+^, and NO signaling assays. It is known that stimulation of the MOR1 leads to the dissociation of the heterotrimeric Gα_i/o _-protein complex, where release of the α subunit results in the inhibition of the adenylyl cyclase/cAMP pathway and release of the βγ subunits inhibits VGCC [15,16]. Therefore, the cellular characterization of cAMP accumulation and intracellular Ca^2+ ^levels were used to assess the functional effects of MOR1K activation [16,27,41-44]. In agreement with an inhibitory function of MOR1, COS1 cells transfected with this isoform demonstrated a significant decrease in forskolin-induced cAMP levels following treatment with 1 μM of morphine. In contrast, COS1 cells transfected with MOR1K did not show a morphine-dependent decrease in forskolin-induced cAMP levels. Instead, they showed a trend towards an increase in cAMP levels (Fig.2A). Furthermore, COS1 cells transfected with MOR1K showed a substantial increase in intracellular Ca^2+ ^release following morphine treatment, which was not observed in MOR1 expressing cells (Fig. 2B). These findings were replicated in Be2C neuroblastoma cells (Fig.2C).

Because VGCC appears to be the primary target underlying the rapid inhibitory effects of opioids[17,18] and morphine stimulation of MOR1K increases intracellular Ca^2+ ^levels in COS1 cells (Fig.2B), we examined the dose-dependent regulation of Ca^2+ ^levels in neuroblastoma BE2C cells transiently transfected with MOR1K (Fig.2C). In BE2C cells, stimulation of MOR1K with morphine produced a robust dose-dependent increase in Ca^2+ ^levels that was blocked by naloxone. A moderate increase in morphine-evoked Ca^2+ ^levels was also observed in Be2C cells transfected with MOR1 or empty vector, however this was likely due to the high endogenous expression of MOR1K in this cell line (Fig.1B). Consistent with this view, the increase in Ca^2+ ^levels in cells transfected with MOR1K was significantly higher than the increases observed in cells transfected with either MOR1 or empty vector, all morphine-dependent Ca^2+ ^increases were sensitive to opioid-receptor blockage with naloxone and MOR1-dependent morphine-evoked increases in Ca^2+ ^were not observed in COS1 cells that do not express endogenous MOR1K (Fig. 1B, 2B).

**Figure 3:**
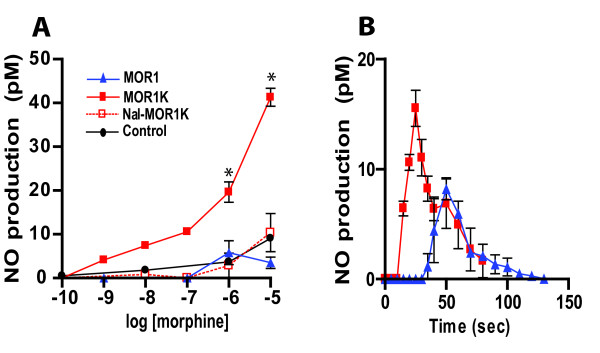
**MOR1K activation stimulates NO production**. Be2C cells were transiently transfected with MOR1K or MOR1 expressing constructs. (A) MOR1K produced robust concentration-dependent increases in the release of NO. (B) The time course associated with morphine-induced (1 μM) NO release was markedly different in response to stimulating MOR1K and MOR1. NO production was blocked by pretreatment with naloxone (0.1 μM) and was significantly different from the respective controls. Data are presented as mean + SEM. *P < 0.05 different from controls.

Because the main cellular response mediated by another reported 6TM isoform, MOR-3, is a morphine-dependent increase in nitric oxide (NO) production [45], we examined the effects of morphine on NO release from immortalized cell lines transfected with MOR1 or MOR1K isoforms (Fig.3). There were marked differences between MOR1 and MOR1K with respect to morphine-induced NO production in transfected Be2C cells. In contrast to MOR1, activation of MOR1K with morphine produced a substantial dose-dependent increase in NO that was blocked by naloxone (Fig. 3A). Also, the time required for NO to reach its maximum level occurred more rapidly in MOR1K relative to MOR1 transfected cells. For MOR1K expressing cells, maximum NO production (16 pM) occurred at ~25 seconds and returned to a baseline at ~80 sec following 1 μM morphine administration. For MOR1 expressing cells, maximum NO production (7.5 pM) occurred at ~50 sec after administration of 1 μM morphine (Fig. 3C). Similar results were obtained in COS1 cells (data not shown). Importantly, morphine-dependent release of NO promotes a reduction in opioid analgesia as well as an increase in analgesic tolerance and OIH in animal models [46-49].

### MOR1K coupling to G-protein complexes

**Figure 4:**
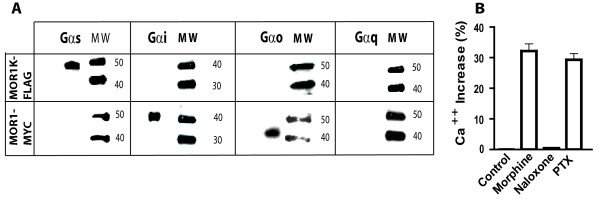
**MOR1K couples to Gα_s_**. COS1 cells were transiently transfected with MYC-tagged MOR1 or FLAG-tagged MOR1K constructs. (A) Iimmunoprecipitation was performed with anti-FLAG (raised in rabbit) and anti-MYC (raised in rabbit) antibodies. Immunoblotting was conducted with specific anti-Gα antibodies as described. The MOR1K-FLAG construct was found to couple to Gα_s_, while the MOR1-MYC construct coupled only to Gα_i/o_. The exposure time for MOR1K-FLAG experiments were ~2 times longer to see band intensities comparable to MOR1-MYC. (B) Consistent with Gα_s _binding to the MOR1K isoform, morphine-mediated increases in Ca^2+ ^levels were not prevented by pre-treatment of MOR1K expressing cells with PTX (100 ng/ml for 15min).

GPCR signaling is initiated by activating heterotrimeric G-protein complexes. Uncoupling of Gα_i_-Gα_o _inhibits AC resulting in decreased cAMP production, while the release of βγ subunits inhibits Ca^2+ ^channels so as to inhibit neural activity. Conversely, uncoupling of Gα_s _and Gα_q _subunits results in increased cAMP production and cellular excitation. Electrophysiological studies of the effects of opioids on isolated nociceptive-like dorsal root ganglion (DRG) neurons provides *in vivo *evidence that the inhibitory effects (e.g. shortening of the Ca^2+^-dependent component of the action potential duration and inhibition of transmitter release) are mediated by Gα_i/o_-dependent pathways. In contrast, the excitatory effects (e.g. prolongation of the action potential duration and stimulation of transmitter release) are mediated by Gα_s _-dependent pathways[11,31]. Since activation of MOR1K results in the intracellular accumulation of Ca^2+ ^and showed a tendency to increase cAMP levels, we examined whether MOR1K couples to Gα_s_, versus Gα_i_. A set of co-immunoprecipitation experiments was conducted to elucidate MOR1K's coupling partner (Fig. 4). As expected, MOR1 co-immuprecipitated with Gα_i _and Gα_o_. In contrast, MOR1K co-immuprecipitated with Gα_s_. These results are consistent with the observation that treatment with pertussis toxin does not block MOR1K-dependent increases in intracellular Ca^2+ ^(Fig. 4). Coupling with Gα_q _was not observed for either MOR1 or MOR1K.

### Molecular and medical implications

Our results have very broad basic molecular and medical implications. First, they significantly contribute to our emerging understanding of the molecular and cellular biology of MOR receptor and GPCRs in general. MOR1K is one of several human and mouse MOR alternatively spliced variants coding for a truncated 6TM receptor lacking an extracellular N-terminal domain and transmembrane domain I [45,50,51]. To date, the functional significance of truncated 6TM MOR receptors has not been examined. The results of our studies are the first to show that activation of this 6TM MOR isoform results in increased production of mediators (Ca^2+ ^and NO) associated with cellular excitation. Furthermore, immunoprecipitation experiments revealed that the MOR1K 6TM receptor couples to Gα_s _rather than Gα_i_. Unlike the stimulation of the MOR1 7TM, which inhibited the production of cAMP, morphine stimulation of the MOR1K 6TM failed to inhibit cAMP production and instead tended to increase cAMP production. The finding that MOR1K receptor couples with Gα_s _suggests that we were not able to fully assess the extent of the cAMP regulation by MOR1K, which will require additional optimization of the cellular conditions. However, the robust effect of morphine on MOR1K-dependent Ca^2+ ^and NO release, in conjunction with a primary role of VGCC in mediating the analgesic effects of opioids [17,18] and the observation that MOR1K couples to Gα_s_, supports the view that MOR1K, and perhaps other 6TM isoforms, function to counteract the cellular actions mediated by canonical 7TM isoforms.

Although there are several examples in other receptor systems where truncated isoforms modulate the activity of [52-56] or display opposite biological activity relative to the canonical receptor [57], the functional significance of this interaction has not been fully appreciated. Because GPCRs are the major targets for therapeutic drugs commonly used in clinical practice, and only a few other 6TM GPCRs isoforms have been reported (e.g., histamine H_3_[55], prostanoid [58] and adrenergic alpha1A [56] receptors), our data open up a new vista for a fuller investigation of the functional importance of 6TM truncated GPCR isoforms in GPCR signaling and drug responses.

The observed intracellular localization of the MOR1K receptor isoform, while relatively rare, is well described for other functional receptor systems. One well known example is the sigma receptor (Sigma-1) that reside primarily at the endoplasmic reticulum and whose ligands include cocaine, (+)-benzomorphans like (+)-pentazocine, (+)N-allyl-normetazocine, and endogenous neurosteroids like progesterone and pregnenolone sulfate [35]. Many pharmacological and physiological actions have been attributed to sigma-1 receptors. These include the regulation of IP3 receptors and calcium signaling at the endoplasmic reticulum, mobilization of cytoskeletal adaptor proteins, modulation of nerve growth factor-induced neurite sprouting, modulation of neurotransmitter release and neuronal firing, modulation of potassium channels as a regulatory subunit, promotion of psychostimulant-induced gene expression, and blockade of spreading depression [59]. Among GPCRs, a well known example of an intracellularly located receptor is GPR30 that is localized to the endoplasmic reticulum, where it specifically binds estrogen. Activation by estrogen results in intracellular calcium mobilization and synthesis of phosphatidylinositol 3,4,5-trisphosphate (IP3) in the nucleus [34]. However, the possibility that MOR1K cell surface expression is very transient or/and requires a chaperone for plasma membrane co-expression [60,61] can not be ruled out.

Finally, our results also suggest that MOR1K may mediate the molecular processes that underlie OIH and possibly pharmacological tolerance commonly observed in response to opioids. Because stimulation of MOR1K results in the production of excitatory mediators such as Ca^2+^, and NO that have been shown to contribute to tolerance and OIH [16,46-49,62], the up-regulation of this isoform or desensitization of the major 7TM MOR1 isoform in response to prolonged opioid administration may result in a change in the balance between 7TM MOR- and 6TM MOR1K-mediated activities and thus dictate the effects of MOR stimulation on physiological processing of nociceptive information. Thus, further investigation of MORK1 expression and function, in conjunction with major MOR1 isoform expression and function under chronic opioid exposure in animal and human models is required.

## Conclusion

In summary, MOR agonists are among the most widely prescribed analgesics for both acute postoperative pain and chronic pain conditions; yet, there are substantial drug-induced side-effects of which we have very limited understanding. Our results provide substantial evidence that the 6TM MOR1K isoform is not just another alternatively-spliced form of *MOR1*, but instead it represents a functional receptor that contributes to the net cellular response of MOR agonists by facilitating excitatory effects. Thus, MOR1K may represent a new molecular target that mediates OIH, and analgesic tolerance. Elucidating the biological and cellular properties of 6TM and 7TM MOR receptor variants may ultimately lead to the identification and development of new classes of opioid analgesics, such as 7TM-selective agonists and/or 6TM-slelective antagonists, that show a high degree of analgesic efficacy with fewer treatment limiting side-effects.

## Methods

### Cloning

MOR1K and MOR1 expression constructs were subcloned into pIRES2-EGFP (Clontech) expression vector using SacI/SacII restriction sites. MOR PCR products were generated using the following primers: for MOR1 TATCGAGCTCGCCACCATG-GACAGCAGCGCTGCCCCCACGAAC and ATCCCCGCGGTTAGGGCAACGGAG-CAGTTTCTGCTTC; for MOR1K ATGCTCTAGATTAGGGCAACGGAGCAGTTT-CTGCTTC and AAGCTTGCCACCATGAAGACTGCCACCAACATCTACATTTTC). The following primers were used to fuse Myc and Flag sequences to 5'end of MOR1 and MOR1K: ATATCGAGCTCGCCACCATGGAGCAAAAGCTCATTTCTGAAGAGGACTTGATGGAACAGAAATTGATCAGCGAGGAAGATCTGATGGACAG and ATATCGAGCTCGCCACCATGGATTACAAGGACGACGATGACAAGGACTATAAAGACGATGACGATAAAATGAAGACTGCCACC, respectively. All constructs were sequence verified.

### Cell culture

Be2C, COS1, SH-SY5Y, HEK293 and H4 cell lines were obtained from ATCC. The cells were grown to 90% confluence and transfected with MOR1K, MOR1 or GFP expression constructs with Lipofectamine 2000 reagent (Invitrogen) according to manufacturer's guidelines.

### RT-PCR analysis

RNA from cell lines was extracted using a Qiagen RNeasy kit. The human tissues RNA samples were purchased from Takara Bio. The 2-3 μg of RNA was treated with TURBO-DNA free (Ambion) and reverse transcribed using SuperScript III Reverse Transcriptase (Invitrogen) kits. The cDNA was amplified with SYBR Green PCR Master mix (Applied Biosystems Inc) using forward and reverse PCR primers using 50°C for 2 min and 95°C for 10 min followed by 40 cycles of 95°C for 15 sec and 60°C for 1 min. 7900HT Fast real-Time PCR system (ABI) was used for measuring RNA transcripts amplification. The same exon 2-specific reverse primer (GCCAGAGCAAGGTTGAAAATG) was used in combination with either exon 1-specific forward primer (CTTCCTGGTCATGTATGTGATTGTC) or exon 13 -specific forward primer (AGTGGTTCCCAGAGTGAAACTGA).

### Confocal microscopy

HEK293 or Be2C cells were fixed with paraformaldehyde (4%) and subsequently permeabilized with Triton X-100 (.003%). Permeabilized cells were stained using Anti-Myc-Tag Antibody (Alexa Fluor 647 Conjugate), Anti-DYDDDDK Tag Antibody (Alexa Fluor 555 Conjugate) (Cell Signaling), Anti-FLAG M2 Antibody Alexa Fluor Conjugate or fluorescent-labeled naloxone (FNAL, Sigma) and coated with Prolong Gold Anti-Fade Reagent (Invitrogen). Images were obtained on an Olympus FV500 confocal laser scanning microscope with excitation wavelengths of 546 nm and 647 nm.

### Flowcytometry analysis

Be2C cells were stained on ice in PBS containing 2% FBS. Fluorescence intensities were acquired using a CyAn ADP high resolution cytometer (DAKO, Fort Collins, CO). Concentration of unlabeled naloxone was maintained at 10 μM throughout the experiment. Dead cells were excluded using forward and side scatter characteristics and at least 2 × 10^4 ^live cells were acquired. The intensity of staining was expressed in arbitrary units of fluorescence. Four independent experiments were performed in triplicate. Mean values were compared using two way ANOVA followed by Tukey's test.

### cAMP assay

For the cAMP accumulation assay, MOR1 or MOR1K expressants were plated on 12 well plates and grown to 90% confluency. On the day of sample preparation, cells were washed with DMEM to remove serum and incubated with serum-free DMEM containing the phosphodiesterase inhibitor IBMX (100 μM)(Sigma) for 30 min. Morphine was then added and cells incubated for a further 15 min. Following this, forskolin FSK (50 μM) was added to the wells and the cells were incubated for 15 min to stimulate cAMP production. DMSO alone was used as a vehicle control. After incubation, reactions were terminated by aspiration of the medium and addition of 0.1 M HCl followed by 20 min incubation at room temperature. After centrifugation of the cell samples at 10,000 × g for 10 min, protein content of the supernatant was assessed and the samples were diluted to protein concentrations of 20 μg/ml. The levels of cAMP were determined using an enzyme immunoassay (EIA) cAMP EIA kit (Cayman Chemical) according to the manufacturer specifications.

### Calcium measurement

Be2C human neuroblastoma cells were grown to near confluence in black 96-well poly-D-lysine coated plates. The cell cultures were grown in DMEM/F12 media. The indicator Fluo-4 NW dye (Invitrogen) was prepared as outlined in manufacturer instructions. 100 μL of Fluo-4 NW dye was added to each well. The plate was then incubated with the lid on at 37°C for 30 minutes, then at room temperature for an additional 30 minutes. The fluorescence was measured using Victror-3 (Perkin Elmer) microplate reader with settings for emission at 515 nm and excitation at 500 nm.

### Nitric oxide (NO) measurement

Cells were grown in DMEM/F12 supplemented with 10% FBS at 37°C. NO release from the transfected and untransfected cell lines was directly measured using an NO-specific amperometric probe (Innovative Instruments). The system was calibrated daily by nitrosothiol donor *S*-nitroso-*N*-acetyl-D,L-penicillamine (World Precision). The amperometric probe was allowed to equilibrate for at least 10 min before being transferred to the well containing the cells. Morphine-stimulated NO release was evaluated in response to increasing morphine concentrations in the range of 10^-5^-10^-9 ^M. Each experiment was repeated four times along with a control (cells transfected with vector alone).

### Co-Immuno precipitation

The cells were lysed using RIPA buffer and centrifuged at maximum RPM for 20 minutes. Supernatants were collected and used for co-IP experiments. Following the overnight incubation on the rotary shaker at 4°C with anti-FLAG (MOR1K) or anti-MYC (MOR1) antibodies, the beads (Pierce) were added, and the samples were incubated for 6 hours at 4°C. The samples were then centrifuged at 14000 rpm for 10 min, the supernatants were discarded, and the beads were re-suspended in 50% RIPA/PBS buffer. The procedure was repeated 3 times. The samples were then boiled and run on 12% SDS-PAGE gels (Invitrogen) and transferred to nitrocellulose membranes (Hybond ECL; GE Healthcare Bio-Sciences). After blocking overnight at 4°C with 5% non-fat dried milk in Tris-buffered saline with Tween 20 (150 mM NaCl, 20 mM Tris-HCl, pH 7.5, 0.1% Tween 20), the membranes were probed with primary antibodies [anti-Gα_s _(K-20): sc-823, anti-Gα_i_ (C-10): sc-262 and anti-Gβ (T-20): sc-378; Santa Cruz Biotechnology, 1:1,000] overnight at 4°C. After washing in TBS-T (three times, 5 min each), the blots were incubated for 4 hours at room temperature with horseradish peroxidase-conjugated secondary antibody (1:15,000; GE Healthcare Bio-Sciences). All antibodies were diluted in blocking buffer. The antibody-antigen complexes were detected using the ECL system (Amersham) and visualized with photosensitive film (Kodak).

## List of abbreviations

OIH: opioid-induced hyperalgesia; MOR: μ opioid receptor; 7TM: 7-transmembrane; 6TM: 6-transmembrane;Ca^2+^: calcium; cAMP: cyclic adenosine monophosphate; FSK: forskolin; IBMX: 3-isobutyl-1-methylxanthine; EIA: Enzyme immune assay; PTX: Pertussis toxin; VGCC: voltage-gated calcium channel; K^+ ^channel: potassium channel; AC: Adenyl cyclase; SNP: single nucleotide polymorphism; *OPRM1*: μ opioid receptor gene; TMH: trans-membrane domain; DRG: dorsal root ganglion; NO: nitric oxide; GPCR: G-protein coupled receptor; IRES: internal ribosome entry site; EGFP: enhanced green fluorescence protein; RIPA buffer: Radioimmunoprecipitation assay buffer; ECL: enhanced chemiluminescence.

## Competing interests

*Financial competing interests*: Luda Diatchenko, William Maixner, Pavel Gris and Josee Gauthier are listed as inventors on a pending patent relating to the content of the manuscript. During the last five years, Drs. Maixner and Diatchenko have received consulting fees from a company they cofounded, Algynomics Inc. This company has the rights to the pending patent in areas related to OPRM1 and work described in this manuscript. Drs. Maixner, Diatchenko, and Nackley are also equity stock holders in Algynomics. Algynomics has not provided funding for the work described in this manuscript. *Non-financial competing interests: *Other than current and recently submitted NIH grant applications there are no other non-financial competing interests

## Authors' contributions

PG participated in the design of the study, carried out the NO and Ca^2+ ^response studies, the, co-IP and labeled naloxone confocal imaging studies, and drafted the figures and manuscript. JG carried out expression studies, and helped draft the figures and manuscript. PC carried out Ca^2+ ^assays. DGG carried out co-IP assays and Western blotting. DG designed and performed flow cytometry experiments. OL designed the expression clones and performed cloning and the sequence alignment. JP carried out cAMP assays. SW carried out the confocal microscopy imaging. AGN participated in its design and coordination and helped draft the manuscript. WM conceived of the study, participated in its design and helped to draft the manuscript. LD conceived the study, participated in its design and coordination, and helped to draft the manuscript. All authors read and approved the final manuscript.
